# The Gut Bacterium *Bacteroides thetaiotaomicron* Influences the Virulence Potential of the Enterohemorrhagic *Escherichia coli* O103:H25

**DOI:** 10.1371/journal.pone.0118140

**Published:** 2015-02-26

**Authors:** Hildegunn Iversen, Toril Lindbäck, Trine M. L’Abée-Lund, Norbert Roos, Marina Aspholm, Lotte Stenfors Arnesen

**Affiliations:** 1 Department of Food Safety and Infection Biology, Norwegian University of Life Sciences, Oslo, Norway; 2 Department of Biosciences, University of Oslo, Oslo, Norway; Texas A&M University, UNITED STATES

## Abstract

Enterohemorrhagic *E*. *coli* (EHEC) is associated with severe gastrointestinal disease. Upon entering the gastrointestinal tract, EHEC is exposed to a fluctuating environment and a myriad of other bacterial species. To establish an infection, EHEC strains have to modulate their gene expression according to the GI tract environment. In order to explore the interspecies interactions between EHEC and an human intestinal commensal, the global gene expression profile was determined of EHEC O103:H25 (EHEC NIPH-11060424) co-cultured with *B*. *thetaiotaomicron* (CCUG 10774) or grown in the presence of spent medium from *B*. *thetaiotaomicron*. Microarray analysis revealed that approximately 1% of the EHEC NIPH-11060424 genes were significantly up-regulated both in co-culture (30 genes) and in the presence of spent medium (44 genes), and that the affected genes differed between the two conditions. In co-culture, genes encoding structural components of the type three secretion system were among the most affected genes with an almost 4-fold up-regulation, while the most affected genes in spent medium were involved in chemotaxis and were more than 3-fold up-regulated. The operons for type three secretion system (TTSS) are located on the Locus of enterocyte effacement (LEE) pathogenicity island, and qPCR showed that genes of all five operons (LEE1-LEE5) were up-regulated. Moreover, an increased adherence to HeLa cells was observed in EHEC NIPH-11060424 exposed to *B*. *thetaiotaomicron*. Expression of *stx2* genes, encoding the main virulence factor of EHEC, was down-regulated in both conditions (co-culture/spent medium). These results show that expression of EHEC genes involved in colonization and virulence is modulated in response to direct interspecies contact between cells, or to diffusible factors released from *B*. *thetaiotaomicron*. Such interspecies interactions could allow the pathogen to recognize its predilection site and modulate its behaviour accordingly, thus increasing the efficiency of colonization of the colon mucosa, facilitating its persistence and increasing its virulence potential.

## Introduction

The human intestinal tract is colonized by a huge number of commensal microbes and the composition and importance of this microbiota in human health and disease have been studied increasingly the last years. It is now known that the intestinal microbiota is central to the health of the host by influencing the metabolism, physiology, nutrition and immune function [1,2]. The intestinal microbiota also plays an important role as a protective barrier against pathogenic microorganisms by competing for nutrition and attachment sites on the epithelium, boosting the host’s gut- and systemic immune response, and producing various antibacterial substances [3]. To overcome this hurdle, enteric pathogens must have mechanisms to interact and cope with the resident microbial community and with numerous host- and environmentally derived stressors that affect their functionality and pathogenic processes. In fact, their pathogenicity is the net result of numerous interactions with either their microbial environment or their host (reviewed in [4,5]).

The dominating bacteria in the distal human gastrointestinal tract are Bacteroidetes (17–60%) and Firmicutes (35–80%) under normal conditions, suggesting that these phyla have important functions in the host [6–8]. Other common phyla in the gastrointestinal tract are the Proteobacteria, Actinobacteria and Euryarchaeota [9]. Bacteria of the *Bacteroidaceae* family are significant contributors to polysaccharide degradation and uptake in the human gut, and members of the *Bacteroides* family produce a high number of glycosyl hydrolases compared to other commensals [10]. *B*. *thetaiotaomicron* cleaves fucose from host glycans, which results in free fucose available in the gut lumen [11]. Some pathogenic *E*. *coli*, e.g. enterohemorrhagic *E*. *coli* (EHEC) O157 carry a fucose-sensing two-component signal transduction system which senses free fucose and this mechanism is involved in the timing of virulence and metabolic gene expression [12]. It has also been shown that *B*. *thetaiotaomicron* (and other commensals) produces an unidentified extracellular molecule, of molecular mass below 3 kDa, which inhibits the production of Shiga toxin 2 (Stx2) in EHEC O157:H7 [13]. However, the overall gene expression profile of an EHEC in co-culture with the predominant human commensal *B*. *thetaiotaomicron* has, to our knowledge, not been explored.

EHEC is associated with increasing numbers of disease outbreaks and sporadic cases of human disease worldwide [14,15]. The disease is characterized by initial diarrhoea, sometimes followed by bloody diarrhoea, and can occasionally progress to the serious, life-threatening condition hemolytic uremic syndrome (HUS) [16]. EHEC disease is characterized by a low infectious dose, which may be associated with elevated acid tolerance [17] and most likely, competitive abilities in order for EHEC to survive in the intestinal environment and colonize the epithelial cells lining the terminal ileum and colon [17–19].

Pathogenesis of EHEC is mediated by multiple mechanisms, but the main virulence factor is the Shiga toxin [20]. The genes encoding Shiga toxin are carried in the genomes of lambdoid bacteriophages, and toxin expression is controlled by a promoter present in the phage genome [21,22]. In contrast to commensal *E*. *coli* strains, which live in the mucus layer, EHEC is found in close contact with the epithelium [23]. Attachment of EHEC to intestinal cells is associated with the destruction of microvilli and the formation of a highly organized cytoskeletal structure termed an attaching and effacing (AE) lesion [24]. Central in these key events is the LEE. LEE is a pathogenicity island encoding proteins which play an important role in initial attachment to enterocytes, and in the translocation of effector proteins (EspD, EspB and Tir) into the host. LEE harbours genes encoding a type three secretion system (TTSS), the adhesin intimin and the intimin receptor Tir (translocated intimin receptor) (reviewed in [24]). The formation of the AE-lesion is an important step in the EHEC infection process, and the structure is made after translocation of Tir into the host cell via the TTSS system [25,26]. The interaction between Tir and intimin modulates multiple host signalling cascades that lead to actin polymerization creating the characteristic AE-lesions [27,28].

Studies regarding adaptive regulation of genes in EHEC have mainly focused on changes in expression of virulence genes under varying growth conditions [29–31], in the presence of eukaryotic cells [32], and in co-cultures with probiotic bacterial species [33,34]. It has also been shown that the intestinal microbiota affects virulence gene expression in EHEC O157:H7 [13]. Although a large number of studies have focused on understanding how single pathogens interact with their host, huge information gaps remain regarding the ecology of the intestinal microbiota and its interactions with pathogenic bacteria. Elucidating these interactions is of importance as it provides knowledge concerning the mechanisms of the pathogens’ ability to persist in their host and cause disease.

In the present study, we investigated the global gene expression profile, using microarray technology and qPCR, of the highly virulent outbreak strain EHEC O103:H25 (EHEC NIPH-11060424) in co-culture or grown in the presence of spent medium from *B*. *thetaiotaomicron* (the type strain—VPI-5482).

## Results

### Growth of EHEC NIPH-11060424 is not affected in co-culture with *B*. *thetaiotaomicron*

**Fig 1:**
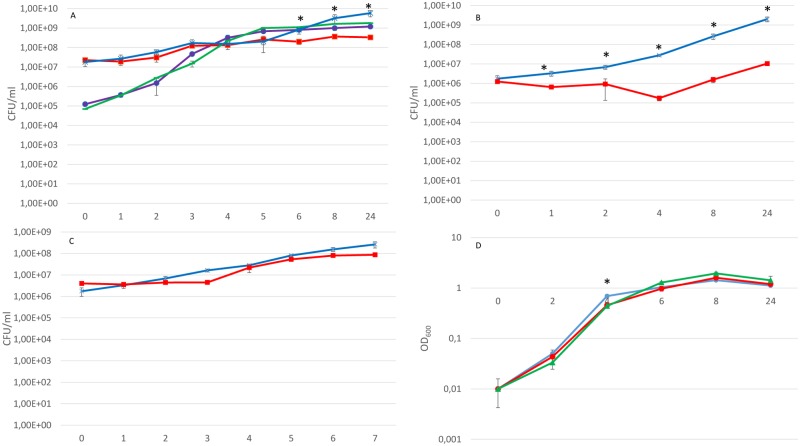
Growth kinetics of EHEC NIPH-11060424 and *B*. *thetaiotaomicron* in co-culture and when grown in spent medium. (A) Growth kinetics of *B*. *thetaiotaomicron* and EHEC NIPH-11060424 grown in pure and co-culture (1:100) under the same conditions as used for microarray analyses. Symbols: purple ● EHEC NIPH-11060424 in co-culture with *B*. *thetaiotaomicron (1:100)*, green *▬* EHEC NIPH-11060424 in pure culture, red ■ *B*. *thetaiotaomicron* in co-culture with EHEC NIPH-11060424 (100:1), blue x *B*. *thetaiotaomicron* in pure culture (B) Kinetics of growth *B*. *thetaiotaomicron* in pure culture and in co-culture with EHEC NIPH-11060424 using equal initial concentrations of the two strains. Symbols: blue x *B*. *thetaiotaomicron* in pure culture, red ■ *B*. *thetaiotaomicron* in co-culture with EHEC NIPH-11060424 (C) Kinetics of growth of *B*. *thetaiotaomicron* in spent medium from EHEC NIPH-11060424. Symbols: red ■ *B*. *thetaiotamicron* in spent medium from EHEC NIPH-11060424, blue x *B*. *thetaiotaomicron* in pure culture (D) Kinetics of growth of EHEC NIPH-11060424 in spent medium from *B*. *thetaiotaomicron* and *B*. *fragilis*. Symbols: red ● EHEC NIPH-11060424 in spent medium from *B*. *thetaiotaomicron*, green ▲EHEC NIPH-11060424 in spent medium from *B*. *fragilis*, blue ● EHEC NIPH-11060424 in pure culture (control). Data represent mean +/- standard error of the mean (SEM) of three independent experiments.

To explore interspecies interactions between *B*. *thetaiotaomicron* and the EHEC strain, the growth kinetics of EHEC NIPH-11060424 grown in pure culture and in co-culture with *B*. *thetaiotaomicron* were examined. As shown in Fig. 1A, the growth kinetics of EHEC NIPH-11060424 was not affected in co-culture with *B*. *thetaiotaomicron* when the ratio of the initial concentrations between the species was 1:100 (EHEC NIPH-11060424: *B*. *thetaiotaomicron*). However, *B*. *thetaiotaomicron* was inhibited when co-cultured with EHEC NIPH-11060424 under the same condition. After 5 hours in co-culture, a growth deceleration-phase in *B*. *thetaiotaomicron* was observed compared to pure *B*. *thetaiotaomicron* culture. After 24 hours in co-culture, the growth of *B*. *thetaiotaomicron* was inhibited 12-fold compared to the growth pattern in pure culture (P < 0.05). When the two strains were co-cultured using equal initial concentrations (10^6^ CFU ml^-1^), the growth inhibitory effect on *B*. *thetaiotaomicron* was more evident and statistically significant growth inhibition was observed in all time intervals except at time point zero (P < 0.05) (Fig. 1B). When *B*. *thetaiotaomicron* was cultured in spent medium from EHEC NIPH-11060424, *B*. *thetaiotaomicron* had a prolonged lag-phase however *B*. *thetaiotaomicron* managed to regain its growth pattern so the biological significance of this extended lag-phase is not known (Fig. 1C). The growth of EHEC NIPH-11060424 was inhibited at time point 4 hours when cultured in spent medium from *B*. *thetaiotaomicron* and *B*. *fragilis*, again the biological significance of this inhibition is difficult to evaluate (Fig. 1D).

### Global gene expression profiles of EHEC NIPH-11060424 in co-culture with *B*. *thetaiotaomicron* and grown in spent medium from *B*. *thetaiotaomicron*

Thirty EHEC NIPH-11060424 genes (0.8% of genes represented on the array) were significantly up-regulated and 25 genes (0.65%) were down-regulated when EHEC NIPH-11060424 was co-cultured with *B*. *thetaiotaomicron*, compared to when cultured alone (at OD = 0.5). In spent medium from *B*. *thetaiotaomicron*, 44 EHEC NIPH-11060424 genes (1.14%) were significantly up-regulated and 37 genes (0.95%) were down-regulated (at OD = 0.5). The location of affected genes was equally distributed throughout the genome.

A selection (>2-fold) of affected genes was assigned to functional groups, based upon gene annotations, using KEGG databases [35,36]. Altered gene expression in three main functional groups was observed: virulence/adhesion, chemotaxis and metabolism (S1, S2 and S3 Files). In co-culture, 13 genes located in the LEE pathogenicity island were up-regulated almost 4-fold compared to pure culture (S1 File). In spent medium, genes involved in chemotaxis were up-regulated more than 3-fold compared to pure culture (S2 File). Thirteen genes involved in metabolism were affected in both groups (co-culture and spent medium at OD = 0.5) and the extent of regulation was similar in the two groups. After induction with Mitomycin C, the *stx2* genes along with associated phage-genes were equally down-regulated in both conditions (S3 File). A brief description of differential expression of genes involved in other functions can be found in S6 File.

### Co-culture with *B*. *thetaiotaomicron* increases expression of LEE genes and increases adherence of EHEC NIPH-11060424 to HeLa cells

Expression of thirteen genes located within the LEE was significantly up-regulated in the microarray analysis when EHEC was co-cultured with *B*. *thetaiotaomicron* (S1 File). In contrast, when EHEC NIPH-11060424 was grown in spent medium from *B*. *thetaiotaomicron*, no up-regulation of the TTSS genes was observed (S2 File). Among the significantly (≥2-fold) up-regulated LEE genes in co-culture were the *Escherichia* secretion components (*escR*, *escS*, *escT* and *escU*) encoding structural parts of the basal body of the TTSS. The Ler (LEE encoded regulator) protein is the major transcriptional regulator of LEE and is encoded by the first gene in the LEE1 operon [37]. The global regulator of *ler* activation (GrlA), which binds to the LEE1 promoter and activates *ler* expression, was also significantly up-regulated.

**Fig 2:**
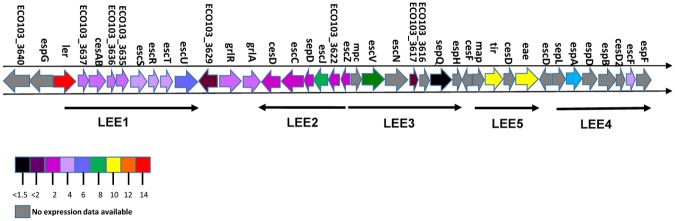
ORF map of the LEE locus to demonstrate differences in gene expression in co-culture compared to pure culture. The ORF map was generated based upon the relative expression ratio of co-culture:pure culture obtained in microarray and qPCR analysis and the graphic presentation of the genetic organization of the LEE pathogenicity island was adapted from Garmendia *et al*. [25].

**Fig 3:**
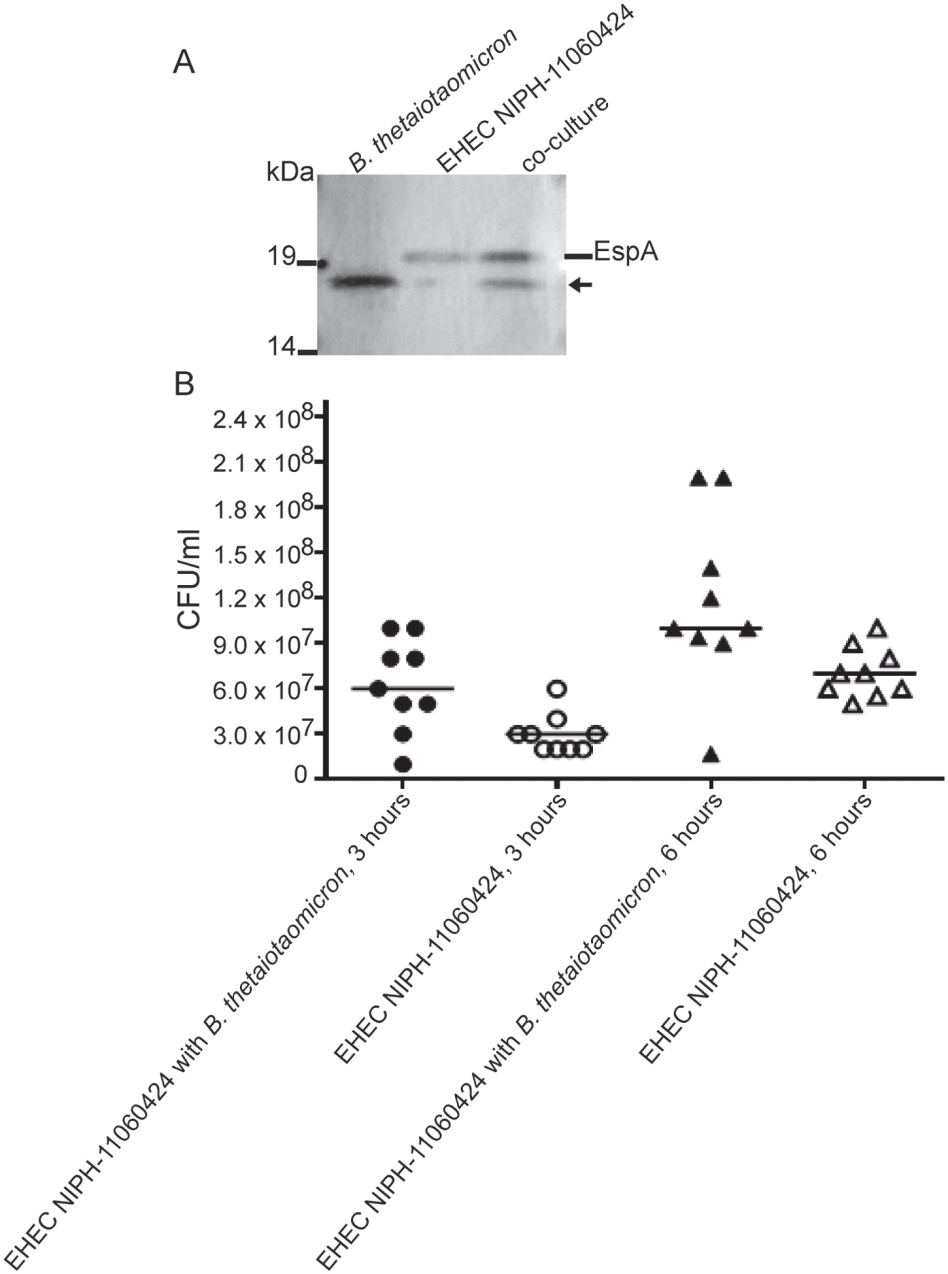
Adherence to HeLa cells, and expression of EspA in co-culture relative to pure culture. (A) Western blot of samples of total cell extracts from pure culture of *B*. *thetaiotaomicron* (lane 1), pure culture of EHEC NIPH-11060424 (lane 2), and *B*. *thetaiotaomicron* EHEC NIPH-11060424 co-culture (lane 3) using anti-EspA monoclonal antibodies. The arrow represents an unknown protein present in *B*. *thetaiotaomicron*. The results shown are representative of three independent biological and technical replicates. (B) The scatter plot shows the adherence of EHEC NIPH-11060424 in co-culture with *B*. *thetaiotaomicron* compared to pure culture of EHEC NIPH-11060424 to HeLa cells after 3 and 6 hours of incubation. The data are representative of three independent experiments with 3 technical replicates (n = 9). The vertical line illustrates the median of each group. The Mann-Whitney non-parametric test was used for comparison of groups. P< 0.05 was considered statistically significant.

The first part of the LEE pathogenicity island (LEE1) encoding the basal body of TTSS is highly conserved between various EHEC strains. However, genes located downstream (LEE2-LEE5) are less conserved and hence prone to weak hybridization in the microarray assay as the array consisted of EHEC O103:H2 12009 probes. Indeed, several of the probes encoding genes located in the LEE2-LEE5 were removed from the dataset due to a mean log2 signal below baseline. Due to these missing microarray data, a selection of genes (*ler*, *escJ*, *escV*, *espA*, *eae*, *tir* and *escF*) representing each of the LEE1-LEE5 operons was analysed by qPCR. The qPCR results revealed that all representative genes from the various LEE-operons were up-regulated significantly in co-culture compared to pure culture (Table 1 and Fig. 2). While differential expression of *ler* was insignificant in the microarray analysis qPCR revealed a significant difference in *ler* expression in co-culture compared to pure culture. The expression of the adhesin protein intimin and its concomitant receptor Tir were up-regulated more than 10-fold, while the genes encoding the major needle subunit of the TTSS EspA and the TTSS needle protein EscF were up-regulated almost 5-fold. In accordance with these results Western blot analysis showed that the level of EspA was increased in co-culture culture relative to pure culture (Fig. 3A). Antiserum against the other TTSS components was unavailable, and hence changes in protein levels by Western blot analysis were not measured. Furthermore, a quantitative adhesion assay using HeLa cells demonstrated significantly increased adhesion properties of co-cultured EHEC NIPH-11060424 relative to EHEC NIPH-11060424 grown in pure culture (3B).

**Table 1:** Comparison of gene expression values obtained with microarray and qPCR.

Gene	Culture condition	Fold change Microarray	Fold change qPCR	Gene description
**LEE-genes**				
*ler*	Co-culture	NA	**13.3**	Transcription regulator
*escU*	Co-culture	**2.6**	**3.1**	TTSS structure protein
*grlA*	Co-culture	**3.2**	**4.3**	positive regulator
*escJ*	Co-culture	**2.3**	**11.3**	TTSS structure protein
*escV*	Co-culture	1.7	**7.3**	translocator EscV
*espA*	Co-culture	NA	**4.8**	Translocon EspA
*eae*	Co-culture	NA	**10.6**	Intimin
*tir*	Co-culture	NA	**10.9**	Translocated intimin receptor
*escF*	Co-culture	NA	**4.4**	TTSS structure protein
**Chemotaxis genes**				
*cheY*	Spent medium	**3.32**	**9.1**	Chemotaxis regulator
*motA*	Spent medium	**2.5**	**5.5**	Flagellar motor protein
*motB*	Spent medium	**2.6**	**6.3**	Flagellar motor protein
*cheZ*	Spent medium	**3.3**	**4.6**	Chemotaxis regulator
*cheB*	Spent medium	**3.06**	**4.5**	Fused chemotaxis regulator
**Metabolic genes**				
*citD*	Co-culture	**4.5**	**37**	citrate lyase acyl carrier subunit
*potE*	Co-culture	**-3.2**	-1.2	putrescine/proton symporter
*glpD*	Co-culture	**-2.95**	-1.4	sn-glycerol-3-phosphate dehydrogenase

NA- not available

A Pearson correlation coefficient of 0.67 was obtained when comparing the fold changes obtained by microarray and qPCR.

Boldface values represent significant changes (P-value ≤ 0.05)

**Fig 4:**
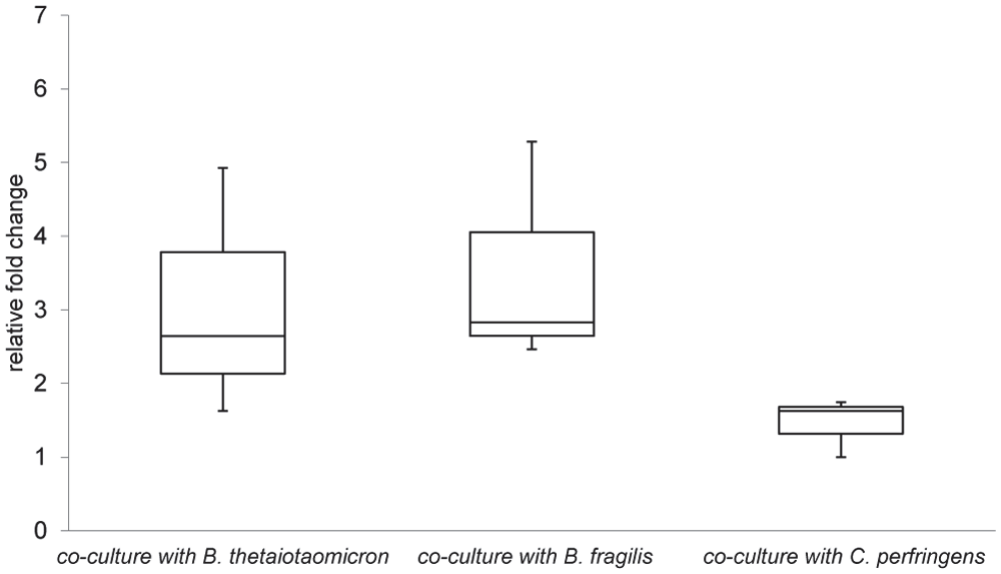
The expression of *escU* determined by qPCR in various conditions. The figure shows relative expression of *escU* when EHEC NIPH-11060424 is grown in co-culture with *B*. *thetaiotaomicron*, *B*. *fragilis* and *C*. *perfringens* compared to growth in pure culture. Boxes show the upper (75%) and the lower (25%) percentiles of the data. Whiskers indicate the highest and the lowest numbers.

To investigate whether the increased expression of TTSS genes was restricted to contact with the *B*. *thetaiotaomicron*, EHEC NIPH-11060424 was co-cultured with two other gastrointestinal species, *Bacteroides fragilis* and *Clostridium perfringens*, and expression of *escU* was determined using qPCR. Interestingly, *escU* was significantly up-regulated in co-culture with *B*. *fragilis* but not with *C*. *perfringens* (Fig. 4).

### *B*. *thetaiotaomicron* repressed *stx2* expression in EHEC NIPH-11060424

**Fig 5:**
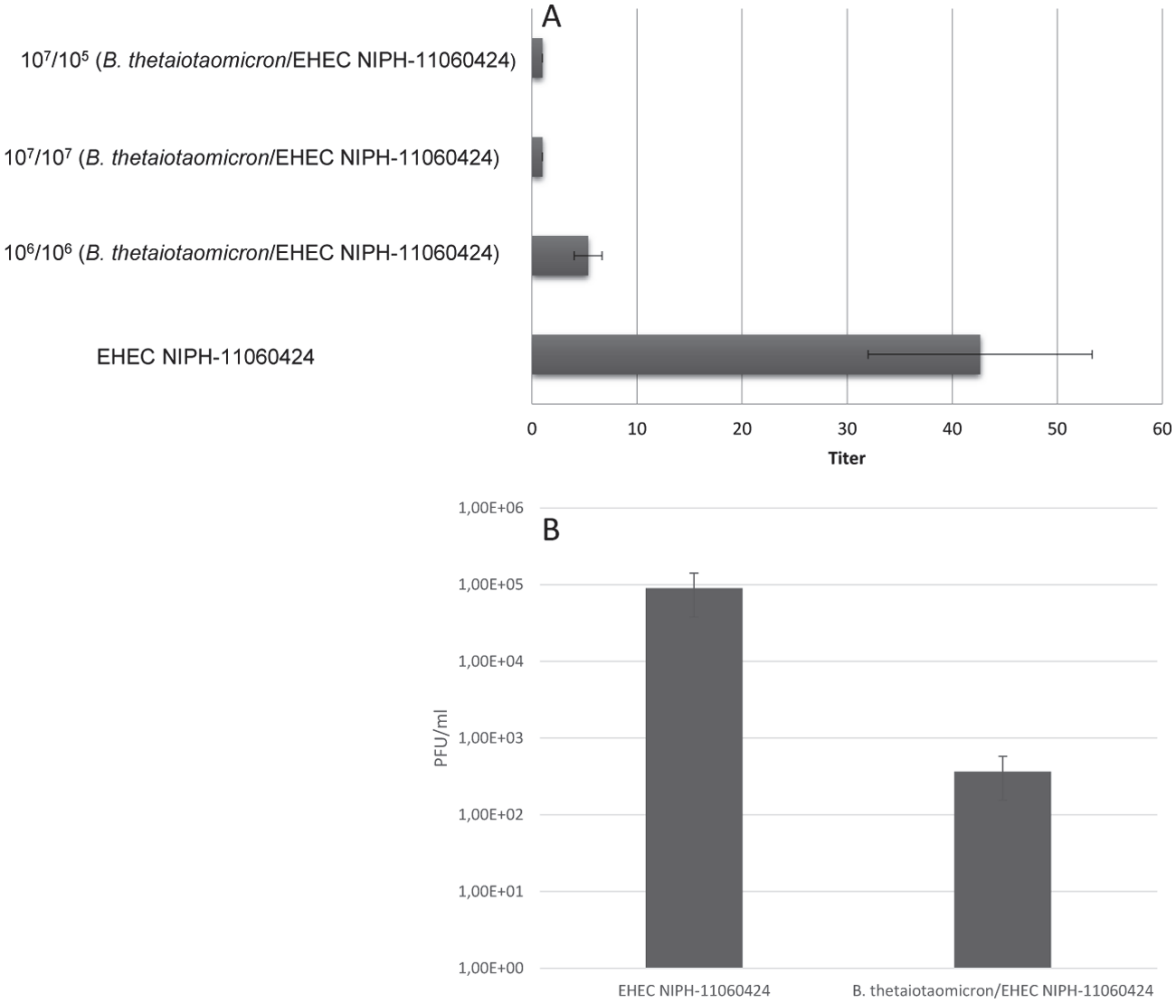
The effect of co-culture on Stx2 production and release of bacteriophages. (A) Stx2 production by EHEC NIPH-11060424 was measured by reverse passive latex agglutination (RPLA) test. EHEC NIPH-11060424 was co-cultured with *B*. *thetaiotaomicron*, using different initial bacterial concentrations. The reciprocal of the highest twofold serial dilution causing latex agglutination was recorded as the titre. Data represent means ± standard errors of the mean (SEM) from three independent experiments. (B) Plaque assay demonstrating production of fewer bacteriophages when EHEC NIPH-11060424 was co-cultured with *B*. *thetaiotaomicron* compared to pure culture.

Microarray revealed a more than 3-fold down-regulation of *stx2* gene expression and various phage-associated genes in mitomycin C induced co-cultures of EHEC NIPH-11060424 and *B*. *thetaiotaomicron*, relative to EHEC NIPH-11060424 in pure culture. A similar down-regulation of *stx2* genes (including other phage-associated genes) was observed when EHEC NIPH-11060424 was cultured in spent medium from *B*. *thetaiotaomicron* (S3 File). The decrease in *stx2* expression levels under these conditions was verified by using the VTEC-RPLA kit (Fig. 5A). Reduced Stx2 production was also observed when EHEC NIPH-11060424 was cultured in spent medium from *B*. *fragilis* and *C*. *perfringens* (S5 File).

To investigate whether the reduced toxin level was due to inhibition of phage production, a plaque assay was performed demonstrating a significant decrease in bacteriophage titre when EHEC NIPH-11060424 was co-cultured with *B*. *thetaiotaomicron*, relative to pure culture (Fig. 5B).

### Altered expression of chemotaxis genes and motility in spent medium

**Fig 6:**
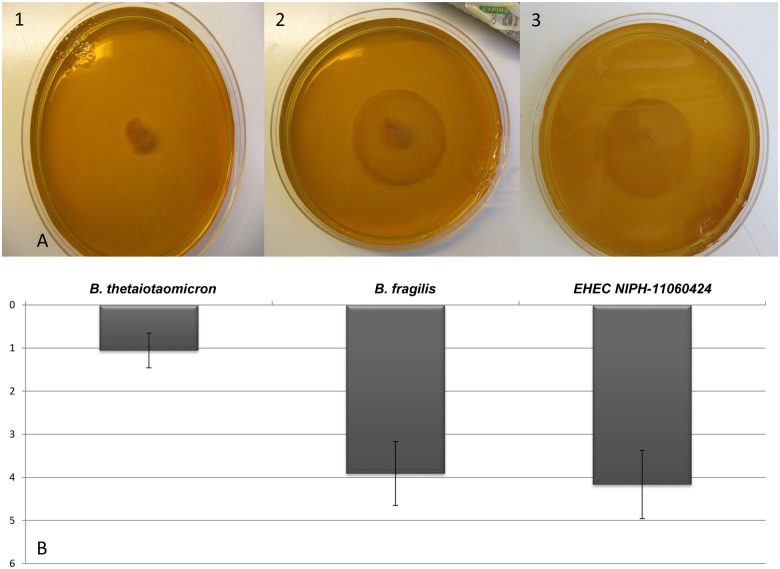
The motility of EHEC NIPH-11060424 in spent medium from various commensals. (A) Motility of EHEC NIPH-11060424 grown in the presence of spent medium from *B*. *thetaiotaomicron* (1), *B*. *fragilis* (2) and in pure culture (filter-sterilized water) (3). (B) The size (cm) of the growth halo in the motility assay upon exposure to spent medium from *B*. *thetaiotaomicron and B*. *fragilis*. Results are given as means of three experiments, with bars showing standard error of the mean (SEM).

When EHEC NIPH-11060424 was grown in spent medium from *B*. *thetaiotaomicron*, a number of chemotaxis and flagellar genes, including *cheY*, *cheA*, *cheZ*, *cheR*, *motB* and *fliS*, were up-regulated (S2 File). In addition, genes encoding chemoreceptors for dipeptides and aspartate (*tap* and *tar*) showed higher expression when EHEC NIPH-11060424 was cultured in spent medium. These chemoreceptors are called methyl-accepting chemotaxis proteins (MCPs) and the binding of an attractant or repellent stimulates cytoplasmic proteins influencing the rotation of the flagella [38]. In concordance, *lrhA*, encoding the transcriptional regulator LrhA, a repressor of flagellar, motility and chemotaxis genes, showed lower expression (S2 File). In the agar-motility assay the presence of spent medium from *B*. *thetaiotaomicron* decreased EHEC motility 4-fold (Fig. 6A (1) and 6B) while the presence of spent medium from *B*. *fragilis* did not affect EHEC motility significantly (Fig. 6A (2) and 6B). Before performing motility assays, EHEC NIPH-11060424 was cultured in spent medium from *B*. *thetaiotaomicron* and *B*. *fragilis* to exclude any growth inhibition exerted by these strains and similar to what was observed earlier, the growth of EHEC NIPH-11060424 was generally not influenced by the presence of spent medium from these two bacterial strains (Fig. 1D).

**Fig 7:**
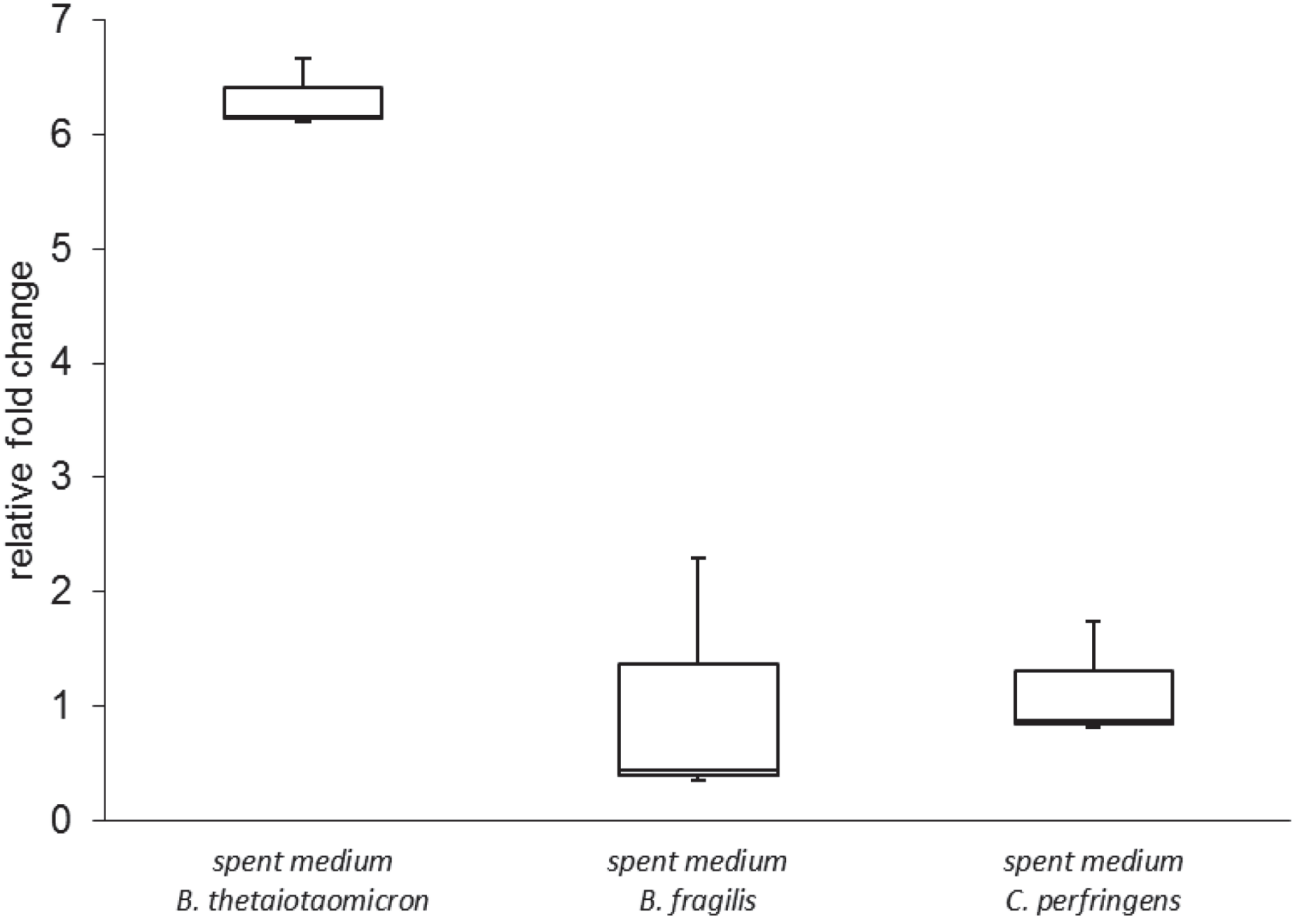
The expression of *cheY* determined by qPCR in various conditions. The figure displays the relative expression of *cheY* when EHEC NIPH-11060424 is cultured in spent medium from *B*. *thetaiotaomicron*, *B*. *fragilis* and *C*. *perfringens* compared to growth in BHI. Boxes show the upper (75%) and the lower (25%) percentiles of the data. Whiskers indicate the highest and the lowest numbers.

To investigate whether chemotaxis genes of EHEC NIPH-11060424 were up-regulated in response to intestinal commensals other than *B*. *thetaiotaomicron*, EHEC NIPH-11060424 was cultured in spent medium from *B*. *fragilis* and *C*. *perfringens*, and gene expression levels of *cheY* were investigated by qPCR. The results revealed that *cheY* was significantly up-regulated when EHEC NIPH-11060424 was cultured in spent medium from *B*. *thetaiotaomicron*. However, no significant change in expression of *cheY* was observed when EHEC NIPH-11060424 was grown in spent medium from *B*. *fragilis* and *C*. *perfringens*, indicating that there is some degree of species specificity in this response (Fig. 7).

### Differently expressed metabolic genes in co-culture and in spent medium

Among the 60 genes that were differently expressed (>2-fold) in co-culture, *citD* was one of the most affected genes, with a 4.5 fold up-regulation. In fact, the whole operon involved in citrate fermentation (*cit* operon) was up-regulated both when EHEC NIPH-11060424 was grown in co-culture (S1 File) and when grown in spent medium from *B*. *thetaiotaomicron* (S2 File). However, the majority of affected metabolic genes (13/19), excluding the *cit* operon, were down-regulated in co-culture compared to pure culture. In the presence of spent medium from *B*. *thetaiotaomicron*, 12 metabolic genes and the *cit* operon were up-regulated, while 15 metabolic genes were down-regulated. The differences in expression profile of metabolic genes expressed in co-culture and in spent medium indicate that EHEC NIPH-11060424 responds differently to direct contact with *B*. *thetaiotaomicron* cells compared to exposure to components present in spent medium from *B*. *thetaiotaomicron*. Six metabolic genes (*speF*, *tnaA*, *dsdA*, *fixC*, *srlA* and *srlE*) were affected in both co-culture and in spent medium, all down-regulated 2-fold (P < 0.05), except *speF*, which was down-regulated 4-fold in spent medium (P < 0.05).

### Confirmation of microarray data with qPCR

To confirm the altered expression patterns observed in the microarray experiments, qPCR was carried out for selected genes representing each affected group. Each assay was performed on three biological replicates and included triplicate PCR of the samples, negative no-template controls, and the reference gene whose expression was similar during all experimental conditions (co-culture/pure culture/spent medium). 12 representative genes were selected as presented in Table 1. The changes in expression levels from the microarray experiments were confirmed, showing a correlation coefficient of ~0.7 (Pearson correlation coefficient) between array results and qPCR (Table 1). The *citD* gene is highly up-regulated both on the microarray and in the qPCR analysis, however the fold change obtained in the microarray for this particular gene, seems for some reason to be underestimated. As the dynamic range of microarray technology is lower compared to that of qPCR, especially after normalization, most genes are expected to have a higher fold-change in the qPCR analysis compared to the microarray analysis [39–41]. Primers for qPCR did not react with cDNA generated from pure cultures of *B*. *thetaiotaomicron* and therefore cross-hybridization was not an issue for these genes.

## Discussion

Among key findings in this study of co-culture of a gut commensal and a major enteric pathogen were that the expression of three main groups of virulence genes and virulence related phenotypes were significantly affected. Among these, the expression of TTSS and adhesion associated genes located within the LEE pathogenicity island were affected by direct contact between *B*. *thetaiotaomicron* and EHEC NIPH-11060424, while the altered expression of several chemotaxis genes was independent of cell-to-cell contact.

The LEE pathogenicity island is arranged into 5 operons termed LEE1-LEE5 consisting of more than 40 genes essential for EPEC and EHEC virulence [26]. The TTSS, formed as a syringe-needle nanomachine able to secrete proteins directly into the host cells, is one of the most complex secretion systems in bacteria [42]. The assembly of the TTSS machinery is a multistep process coordinated by a sequential up-regulation of the TTSS genes [25]. The basal anchoring structure (syringe) is built first and serves as a secretion machine for the needle components [25]. EspA has a dual role in this process, forming filamentous structures involved in initial adhesion of bacteria to the host cell and at the same time building the translocation apparatus for Tir and other effector proteins into the host cell [43].

According to the microarray results, genes within LEE1 and LEE2 encoding the basal body of the TTSS and the regulators GrlA and GrlR, were up-regulated in co-culture compared to pure culture. However, qPCR analysis revealed that all LEE operons were affected in co-culture compared to pure culture, suggesting that the whole LEE pathogenicity island was activated in response to contact with *B*. *thetaiotaomicron* (Fig. 2). Several of the regulators of LEE genes were up-regulated in co-culture including GrlA, GrlR and the major regulator Ler. Ler positively regulates the expression of LEE2-LEE5 and negatively regulates LEE1 [26,44]. The *grlA* and *grlR* constitute a transcriptional unit encoding GrlA (positive regulator) and GrlR (negative regulator) for LEE1 [45]. The increase of the EspA protein in co-culture compared to pure culture and the concomitantly increased adhesion on HeLa cells indicate that contact with *B*. *thetaiotaomicron* in fact increases the virulence potential of the EHEC-strain.

The regulation of LEE gene expression is complex, as it is receptive to several environmental signals, including population status (via quorum sensing), temperature, nutrients and physiological state of cell (growth phase) [46–48]. Previous studies have shown that some EPEC- and EHEC-strains use fucose to regulate the virulence via a two-component fucose-sensing system (FusKR) [12,49]. *B*. *thetaiotaomicron* cleaves fucose from mucin in the gastrointestinal tract resulting in increased fucose availability in the mucus layer [11]. Increased fucose levels lead to repression of LEE-encoded genes in some strains. This fucose-sensing system is exclusively found in the O157:H7-serotype and its progenitor EPEC O55:H7 serotype [12]. The specialised fucose-sensing genes were not found in the genome of EHEC NIPH-11060424, which supports the fact that differences exist between O157 and non-O157-strains. Some EHEC strains thus suppress expression of LEE-genes when present in the mucus layer with *B*. *thetaiotaomicron* [12,49], while the results of the present study show that EHEC increases LEE-gene expression when co-cultured with *B*. *thetaiotaomicron*. This illustrates the fine-tuned balance between expression and repression of genes. The suppression of LEE-genes when present in the mucus-layer perhaps prevents these specific EHEC-strains spending energy expressing virulence in a location where virulence is not necessary. It has not yet been described if, or how, non-O157 EHEC strains may use strategies to conserve energy in the human gut. Non-O157 EHEC strains have a different evolutionary history compared to O157 and represent a heterogeneous group consisting of strains with varying virulence potential [14,50,51].

LEE gene expression in an EHEC-strain of serotype O157:H7 has been demonstrated to increase in a gluconeogenic environment. The transcription factors, KdpE involved in general bacterial homeostasis and the catabolite repressor/activator protein Cra, were demonstrated to be involved in this glucose-regulated process of LEE-gene expression [52]. According to our microarray, no significant differences in the expression of *kdpE* or *cra* genes were observed in our O103:H25 strain when comparing the expression of these genes in co-culture to pure culture. Njoroge *et al*. also demonstrate increased expression of the type three secretion protein EspA when EHEC O157:H7 is co-cultured with *B*. *thetaiotaomicron* [52]. The authors hypothesize that the increased expression could be a result of quorum sensing (QS) as previous studies have demonstrated the production of autoinducers by the intestinal microbiota [53].

Bacteria have several communication systems, allowing them to sense the presence of potential competitors or partners [54,55]. QS is used by bacteria to modulate gene expression patterns based upon population density. The process is mediated by diffusion of signal molecules (auto-inducers) that bind to appropriate receptors on target bacteria [55,56]. We observed no alteration in expression of any of the known QS genes (*qseC* and *qseB*) involved in LEE regulation in EHEC-strains, in co-culture compared to pure culture. Since the EHEC cell density was similar under both conditions, this was not unexpected. It has been demonstrated that quorum sensing signal molecules produced by EHEC and commensal *E*. *coli* influence expression of LEE-genes and it has been suggested that other intestinal bacteria influence expression of LEE-genes as well [53,57,58]. However, since the LEE-genes were not affected when EHEC NIPH-11060424 was cultured in the presence of spent medium from *B*. *thetaiotaomicron*, the up-regulation of LEE-genes in this experiment is most likely not a result of QS.

Recently, contact-dependent signaling systems utilized by bacteria have been found. These systems are used in both intra- and inter-species signaling involving symbiotic and antagonistic interactions [59,60]. An intra-species contact-dependent growth inhibition (CDI) mechanism (type V secretion system) has been described in *E*. *coli*, where the growth of one *E*. *coli* strain is suppressed when it comes in contact with another *E*. *coli* strain [61]. Notably, increased expression of LEE genes was observed only in co-culture, and not when EHEC NIPH-11060424 was grown in spent medium from the *Bacteroides* strain, suggesting that cell to cell contact, or increased local concentration of signal compound(s) provided by *Bacteroides* in close proximity, might trigger the enhanced LEE-gene expression. Interestingly, expression of *escU* was also significantly up-regulated when EHEC NIPH-11060424 was co-cultured with *B*. *fragilis* but not in co-culture with *C*. *perfringens*, indicating that the observed effect on TTSS gene transcription may only be triggered by specific bacterial species. The increase in the expression of TTSS genes in a confined bacterial co-culture was surprising, as the TTSS is currently known to secrete bacterial effectors into eukaryotic cells. Based upon these findings we propose that the interaction with *B*. *thetaiotaomicron* may act as a niche specific signal, priming EHEC for increased adherence to enterocytes and subsequent efficient colonization of its host.

The chemotactic behaviour of bacteria relies on chemosensory adjustment of the activity of the flagellar motor (tumbling/smooth swimming) [62]. The overall direction of movement is determined by whether repellents or attractants are present [63]. The default state of *E*. *coli* is smooth swimming achieved by counter-clockwise rotation of flagella [64]. The cytoplasmic response regulator CheY monitors the direction of flagellar rotation and depending on its phosphorylation state, can either be an activator or inhibitor of smooth swimming [38]. Overall, we observed that major parts of the chemotactic apparatus, from chemo-sensing to motility, were transcriptionally up-regulated by spent medium from *B*. *thetaiotaomicron* compared to pure cultures. Phosphorylated CheY interacts with flagellar rotation causing an increase in tumbling activity (increased clockwise bias) thus inhibiting the motility of EHEC NIPH-11060424 when grown in spent medium from *B*. *thetaiotaomicron*. These findings indicate that *B*. *thetaiotaomicron* might secrete motility inhibiting factor(s). Spent medium from *B*. *fragilis*, another intestinal member of the *Bacteroidaceae* family did not decrease the motility of the EHEC-strain, suggesting that the effect is species specific. Interestingly, the increased expression of motility and chemotaxis genes was not seen in co-cultures of EHEC NIPH-11060424 and *B*. *thetaiotaomicron* but occurred only in the presence of spent medium. Flagella are believed to be important in the early stages of infection contributing to the localization of EHEC close to the epithelial surface [65,66]. However, the regulation of both LEE-genes and flagellar genes should be strict as the simultaneous expression of both groups of genes could impede adhesion [67]. Therefore, the different expression profiles of LEE and chemotaxis genes in co-culture compared to spent medium seen in our study, might relate to a temporal control of virulence factors during EHEC’s positioning to closer contact with the epithelium and a changed closeness with *B*. *thetaiotaomicron*.

The genes encoding the Stx2 toxin are carried by a bacteriophage and thus, expression of the toxin genes is controlled by the bacteriophage itself [22]. Prophage induction, Stx2 production and subsequent cell lysis for release of toxin are believed to occur in the intestine as a result of innate immune effectors working as inducers (e.g. neutrophils producing H_2_O_2_) [68]. In this study mitomycin C (MMC) was used as inducing agent in both co-culture and spent medium experiments. Even after MMC induction, transcription of *stx2* and a number of regulatory bacteriophage genes were strongly repressed in both co-culture with *B*. *thetaiotaomicron* and in spent medium compared to pure EHEC NIPH-11060424 culture. The reduced Stx2 level and phage number measured in co-culture and in spent medium supported the microarray results. The observed decrease in *stx2* transcription also occurred when EHEC NIPH-11060424 was grown in spent medium from the intestinal commensals *B*. *fragilis and C*. *perfringens* (S5 File). These data are in compliance with results presented by De Sablet and co-workers, showing that *B*. *thetaiotaomicron* and other commensals in the gastrointestinal tract produced an unidentified substance with inhibitory effect on Stx2 production in EHEC O157:H7 [13]. However, our data show that the repression of Shiga toxin production also takes place in EHEC of other serotypes than O157:H7. Together, the present and previous findings indicate that the suppression of prophage induction, leading to reduced toxin levels, might be a result of a more universal mechanism, not specific to *B*. *thetaiotamicron*. Considering that intestinal commensals are exposed to numerous bacteriophages in the gastrointestinal tract [69], a system which supresses prophage induction would most likely be beneficial for maintenance of the microbial community.

The expression of a large number of metabolic genes in EHEC was affected both by co-culture and growth in the presence of spent medium from *B*. *thetaiotaomicron*. The competition for nutritional resources, both quantitatively and qualitatively, is a central point in bacterial relations [70], and therefore, the observed effect on metabolic gene expression in co-culture is not surprising. The predominant intestinal bacterium *B*. *thetaiotaomicron* is a primary fermenter thus its activity in the gut will affect the rest of the microbial community [11]. Most metabolic genes were down-regulated in co-culture apart from the *cit* genes and malate dehydrogenase (*mdh*, up-regulated nearly 2-fold). The *cit*-operon is involved in catabolism of citrate under anaerobic conditions, and is important for anaerobic growth [71]. A similar change in expression of citrate metabolic genes have previously been observed by Nouaille *et al*. [72], who reported an up-regulation of the *cit* operon in *Staphylococcus aureus* co-cultured with *Lactococcus*. The *cit* operon was also up-regulated when EHEC NIPH-11060424 was grown in the presence of spent medium from *B*. *thetaiotaomicron*. As EHEC growth is not attenuated by the presence of *B*. *thetaiotaomicron*, or by its secreted components, the resources needed for growth are obviously met. And yet, in co-culture as well as in spent medium from *B*. *thetaiotaomicron*, EHEC NIPH-11060424 changes expression of several metabolic genes, some similarly and some differentially between the two conditions, probably indicating a metabolic modulation in response to the changed conditions.

## Conclusions

In summary, the expression of EHEC NIPH-11060424 genes involved in metabolism, colonization and virulence is modulated in response to direct contact with *B*. *thetaiotaomicron* and to soluble factors released from *B*. *thetaiotaomicron*. In the presence of spent medium from *B*. *thetaiotaomicron*, a number of chemotaxis and flagellar genes were up-regulated and a decrease in motility was observed. The expression of Stx phage genes, including the Shiga toxin (Stx) genes, was down-regulated in mitomycin C induced co-culture/spent medium and accordingly the levels of Stx production and phage release were decreased. Genes encoding the TTSS and other factors involved in adherence to host cells were up-regulated in direct contact with *B*. *thetaiotaomicron*. We also show that direct contact with *B*. *thetaiotaomicron* leads to increased expression of the TTSS protein EspA and increased adhesion to epithelial cells. Based on our findings, we propose that direct contact with *B*. *thetaiotaomicron* could function as a niche specific signal that primes EHEC for a more efficient interaction with the host cells thus increasing its virulence potential.

## Methods

### Bacterial strains

The bacterial strains used in this study are listed in Table 2.

**Table 2:** Bacterial strains used in this study.

Bacterial strain	Serotype/ID	Source	Reference
EHEC NIPH-11060424^a^	O103:H25	EHEC	[90]
EHEC 12009	O103:H2	EHEC	[50]
*B*. *thetaiotaomicron*		CCUG 10774^b^	[91]
*B*. *fragilis*		Isolated from horse	This study
*C*. *perfringens*		DSM756	[92]
DH5α *E*. *coli*		Laboratory strain	[89]

^a^Synonym with NVH-734

^b^Also designated VPI-5482

### Growth conditions

In co-culture experiments EHEC NIPH-11060424 and *B*. *thetaiotaomicron* (CFU ratio 1:100) were grown in modified BHI (BHI (OXOID, UK) with added yeast extract 5 g l^-1^, menadione 1 mg l^-1^ and haemin, 5 mg l^-1^ anaerobically at 37°C [73]. Anaerobic conditions were achieved using an anaerobic work station (Whitley A35 Anaerobic Workstation, Don Whitley Scientific, West Yorkshire, UK). The pure EHEC NIPH-11060424 and *B*. *thetaiotaomicron* cultures were set up under identical conditions. *B*. *thetaiotaomicron* was cultured in modified BHI in an anaerobic atmosphere at 37°C for 24 hours followed by centrifugation at 4500 RPM for 15 minutes and the spent medium was filter sterilized through 0.2 μm filters (Minisart, Sartiorius Stedim Biotech, Goettingen, Germany). For the experiments in spent medium, EHEC NIPH-11060424 was grown in modified BHI supplemented with spent medium from the *B*. *thetaiotaomicron* cultures harvested at 24 hours (1 volume of supernatant:1 volume of 2x modified BHI). For reference, EHEC NIPH-11060424 was grown as pure culture in modified BHI under identical conditions. The pH in spent medium, used for culturing of EHEC, was checked and adjusted to 7 when necessary. For both co-culturing and growth in the presence of spent medium, sample collection was performed at two time points: mid-logarithmic phase (OD = 0.5) and 3 hours after induction with mitomycin C (MMC). The induction with MMC was performed to simulate phage induction in the gastrointestinal tract. Hydrogen peroxide released by neutrophils is believed to activate the bacterial SOS-response subsequently triggering Shiga toxin production [68]. Withdrawn samples were mixed with methanol (500 μl culture/ 500 μl methanol) and kept at -80°C before isolation of RNA. For each condition, three independent biological replicates were established (overview of experiment workflow, see Table 3).

**Table 3:** Overview workflow for microarray experiment.

Culture condition	CFU Ratio	Sample time point experiment	Induction	Biological replicates
Pure culture EHEC NIPH-11060424	NA	mid-logarithmic phase (OD = 0.5)	No	>3
Co-culture EHEC NIPH-11060424/*B*. *thetaiotaomicron*)	1/100	mid-logarithmic phase (OD = 0.5)	No	3
Pure culture EHEC NIPH-11060424 in spent medium from *B*. *thetaiotaomicron*	NA	mid-logarithmic phase (OD = 0.5)	No	3
Induced pure culture EHEC NIPH-11060424	NA	3 hours after induction	MMC	3
Induced co-culture EHEC NIPH-11060424/*B*. *thetaiotaomicron*	1/100	3 hours after induction	MMC	3
Induced culture EHEC NIPH-11060424 in spent medium from *B*. *thetaiotaomicron*	NA	3 hours after induction	MMC	3

To investigate whether differences in gene expression observed in the microarray experiments were also induced by other commensals in the human colon, EHEC NIPH-11060424 was co-cultured with or grown in spent medium from *B*. *fragilis* and *C*. *perfringens*. *B*. *fragilis* was chosen as it is a close relative of *B*. *thetaiotaomicron*. *C*. *perfringens* was selected as it is not related to *B*. *thetaiotaomicron*, it is a Gram-positive bacteria and a representative for the Firmicutes, which is the other main phyla present in the human intestine. The gene expression of LEE genes was represented by measuring the *escU* expression when *B*. *fragilis* and *C*. *perfringens* were co-cultured with EHEC NIPH-11060424. The chemotaxis gene *cheY* was chosen to investigate whether spent medium from other bacteria exerted the same effect as spent medium from *B*. *thetaiotaomicron* on chemotaxis/motility. The same protocol as described for *B*. *thetaiotaomicron* was used for qPCR.

### Growth conditions in growth kinetic experiments

For the growth kinetic experiments with EHEC NIPH-11060424 and *B*. *thetaiotaomicron*, a CFU ratio of 1:100 and 1:1 between the two species was used. In order to quantify viable bacterial cells from pure cultures and co-cultures, bacteria were enumerated on the basis of CFU ml^-1^ on LB-agar and Bacteroides Bile Esculin Agar (BBE-agar, Becton, Dickinson and company, Maryland, USA) at serial time points. The LB-agar was incubated aerobically to ensure growth of EHEC and BBE-agar was incubated anaerobically to ensure growth of *B*. *thetaiotaomicron*. Furthermore, growth kinetics was determined for EHEC grown in spent medium from *B*. *thetaiotaomicron* and *B*. *fragilis* as described above. For these latter experiments growth kinetics were determined by measuring optical density (OD) at 600 nm. All experiments were performed independently three times.

### RNA isolation and cDNA synthesis

Total RNA was extracted using a Purelink RNA mini kit (Life technologies, Carlsbad, California). DNA was removed using the Turbo DNA-free kit (Invitrogen) according to the manufacturer’s instructions. RNA quantity (A_260_) and purity (A_260/280_) were measured in a NanoDrop 1000 spectrophotometer (Thermo Fisher Scientific). RNA quality was determined using Agilent 2100 bioanalyzer.

cDNA for microarray experiments was synthesized according to a recommended protocol from NimbleGen (NimbleGenUser’s Guide) and was performed at the Microarray resource center in Tromsø [74]. For labeling of the cDNA samples, the NimbleGen protocol was followed using direct Cy3-cDNA labelling. For qPCR, cDNA was synthesized from 500 ng RNA using a high-capacity cDNA reverse transcription (RT) kit (Applied Biosystems) according to the manufacturer’s instructions in 20 μl reactions.

### Design of microarray

Changes in gene expression of EHEC NIPH-11060424 in response to *B*. *thetaiotaomicron* (co-culture) or secreted products from *B*. *thetaiotaomicron* (spent medium) were investigated using microarray. Since the complete genome sequence of EHEC NIPH-11060424 was not available at the onset of the study, EHEC O103:H2 strain 12009 [50] was chosen as the template for the array design. An *in silico* comparison was performed between the EHEC NIPH-11060424 strain and EHEC 12009, demonstrating a very high degree of similiarity [75]. A custom-made Nimblegen GeneChip containing 91% (5054/5541) of the open reading frames (ORFs) of *E*. *coli* 12009 was made for this experiment (www.nimblegen.com). Additionally, an *in silico* genome comparison of *B*. *thetaiotaomicron* (VPI-5482) and EHEC 12009 was performed to determine the genetic relatedness between the species, revealing a low genetic similarity. RNA from *B*. *thetaiotaomicron* in pure culture was isolated and converted into cDNA along with the other samples to investigate the possibility of cross-hybridization. No hybridization was observed for samples obtained from pure cultures of *B*. *thetaiotaomicron* confirming that cross-hybridization of genes belonging to *B*. *thetaiotaomicron* is most likely not an issue.

The array design was a 12-plex custom design with 135K probes. Nine probes were selected per transcript and a total of 43838 probes was produced, including 3 replicates per probe. The empty space was filled with random (negative) probes.

### Hybridization and data analysis of microarray

The hybridizations of the mRNA samples were performed at the Microarray resource center in Tromsø, Norway (MRCT). The arrays were read with a GenePix 4000B scanner. The array data were processed using the R statistical language Bioconductor oligo package [76,77]. The oligo package Robust Multichip Average (RMA) normalization algorithm was used. RMA method was recommended by the manufacturer. RMA normalization included background subtraction, quantile normalization and summarization. To further reduce technical variations the ComBat function of the Bioconductor Surrogate Variable Analysis (SVA) was applied [78,79]. Probes with a mean log2 signal under 8 were removed from the dataset (23% of the probes). Comparison was carried out using the Bioconductor linear models for microarray data (Limma) package [80]. Normalization and comparison R scripts are available upon request. A fold-change ≥2 was set as the threshold for differential expression. The gene expression data are accessible in the NCBI Gene Expression Omnibus [81] through GEO Series accession number GSE44790.

### Gene expression changes measured by q-PCR

mRNA levels for selected genes relative to *gapA* (glyceraldehyde-3-phosphate dehydrogenase) were determined by real-time PCR (qPCR). Five microliters of a 1:100 dilution of the cDNA reaction were used as template for qPCR amplification in 25 μl final volumes containing 12.5 μl of Power SYBRgreen PCR master mix with premixed ROX (Invitrogen) and 200 nM of each primer. Primer pairs were designed using Primer3plus [82]. The primers used for qPCR are listed in supplementory materials (Additonal S4 file). qPCR amplification was performed using a StepOne system (Applied Biosystems). The thermal cycling conditions were 10 min at 95°C followed by 40 cycles of 15 s at 95°C and 1 min at 60°C. Fluorescence was monitored during each extension phase, and a melting curve analysis was performed after each run to confirm the amplification of specific transcripts. Again, to reveal any cross-hybridization, a selection of significantly up-regulated or down-regulated genes (from array) was tested for cross-hybridization with selected qPCR primers on pure cultures of *B*. *thetaiotaomicron*.

Changes in gene expression are presented as the differences between treated EHEC NIPH-11060424 in co-culture or in spent medium from various commensals compared to untreated controls. The slope of the standard curve and PCR efficiency for each primer pair were determined by amplifying serial dilutions of the target sequence (S4 File). The results were analyzed using Pfaffl method in combination with the Relative Expression Software Tool (REST) 2009 [83,84].

### SDS-PAGE and immunoblotting

Whole cell lysates for immunoblotting were made from equal numbers of EHEC NIPH-11060424 cells from pure culture and co-culture with *B*. *thetaiotaomicron*. Whole cell lysate was also prepared for *B*. *thetaiotaomicron* in pure culture. Samples were taken at OD_600_ = 0.5, and Procedures for SDS-PAGE and immunoblotting are described in [85]. The antiserum used to detect EspA is a monoclonal antibody directed against the EspA protein (tcgBIOMICS, Aachen, Germany). The primary antibody was used at 1:1000 dilution. Anti-mouse IgG, biotinylated whole antibody (from goat, GE Healthcare, UK) was used as secondary antibody. The result is representative of three independent biological and technical replicates.

### In vitro adherence assays

Analysis of EHEC NIPH-11060424 binding to HeLa cells was performed as described previously [86]. Briefly, HeLa cells were cultured and propagated in MEM with 10% fetal bovine serum according to standard protocols. Prior to the assay, 24-well tissue culture plates were prepared, with HeLa cells cultured at 37°C in 5% CO_2_ for 48 hours and then washed 3 times in 1 ml PBS. The bacterial test cultures were grown anaerobically at 37°C until mid-logarithmic phase (OD = 0.5) and 100 μl of 10^7^ CFU were added to each well of HeLa cells, and incubated for 3 hours and 6 hours at 37°C in a 5% CO_2_ environment. Unbound bacterial cells were removed by washing the wells three times with PBS. The HeLa cells were then lysed, using 0.1% TritonX, followed by preparation of serial dilutions of cell suspension onto LB plates. The plates were incubated aerobically for 24 hours at 37°C and the CFU representing bacteria bound to HeLa cells was determined.

### Semi-quantification of Stx2 levels by VTEC-RPLA kit

VTEC RPLA-toxin detection kit (Oxoid Limited, Basingstoke, UK) was used to determine Stx2 expression levels. The assay was performed according to the manufacturer’s instruction. The amount of sample in each test well was reduced 2-fold at each dilution. The reciprocal of the highest dilution causing latex agglutination was verified as the titre.

### Plaque assay

To compare phage production in co-culture (EHEC NIPH-11060424 and *B*. *thetaiotaomicron*) and pure culture (EHEC NIPH-11060424), the amount of bacteriophages was examined 24 hours after induction with MMC as described by previously [87]. The induced cultures were centrifuged for 10 minutes at 3900 x g and the supernatant was sterilized using 0.2 μm filters (Minisart, Sartiorius Stedim Biotech). In order to remove possible colicins, trypsin was added to the filter-sterilized culture supernatant to a final concentration of 0.1 mg mL^-1^, and incubated for 1 hour at 37°C [88]. The presence of bacteriophages was confirmed by a plaque assay [89]. Briefly, 100 μl of trypsinated phage filtrate was mixed with 900 μl of DH5α culture (OD ~ 0.3) and 3 ml LB soft agar (0.7%) containing (10 mM) CaCl_2_ and poured onto a LB-agar plate. Plaques were counted after overnight incubation at 37°C.

### Motility assay

Motility assays were performed as described previously [86] with minor modifications. Motility agar plates were prepared by mixing one volume spent medium from *B*. *thetaiotaomicron* and *B*. *fragilis* with one volume 2XBHI and agar to a final concentration of 0.3%. Overnight cultures of EHEC NIPH-11060424 were inoculated into modified BHI-medium and grown into mid-exponential phase (OD = 0.5) at 37°C in an anaerobic work station. Five microliters of the culture were placed in the middle of each motility plate. The diameter of the motility halos was measured after 16 hours incubation at 37°C under anaerobic atmosphere. Three motility plates were used for each condition and the experiment was repeated with three independent cultures.

### Statistics

For the growth kinetic data, a t-test was done using SigmaPlot (Systat Software, San Jose, CA). A P-value <0.05 was considered statistically significant.

For the adhesion assays, the Mann-Whitney non-parametric test was used for comparison of groups (Graphpad prism). A P-value <0.05 was considered statistically significant.

Data analysis for qPCR was carried out with the Relative Expression Software Tool (REST) 2009 using the pairwise fixed randomization test. A P-value <0.05 was considered statistically significant.

## Supporting Information

S1 File Relative change in expression of genes of EHEC NIPH-11060424 (co-culture versus pure culture). Summary of changes in expression of selected categories of genes in microarray analysis of EHEC NIPH-11060424 in co-culture with *B*. *thetaiotaomicron* relative to EHEC NIPH-11060424 in pure culture.
(DOCX)

S2 File Relative change in expression of genes of EHEC NIPH-11060424 (spent medium versus pure culture). Summary of changes in expression of genes of selected categories in EHEC NIPH-11060424 cultured in spent medium from *B*. *thetaiotaomicron* relative to pure culture.
(DOCX)

S3 File Relative change in expression of genes of EHEC NIPH-11060424 (co-culture and spent medium after induction). Summary of changes in expression genes in microarray analysis of EHEC NIPH-11060424 in co-culture with *B*. *thetaiotaomicron* and spent medium relative to when EHEC NIPH-11060424 is cultured alone (3 hours after induction with MMC).
(DOCX)

S4 File Overview of primers used for qPCR, (DOCX)

S5 File Inhibition of Stx2 production when EHEC NIPH-11060424 is cultured in spent medium from *C*. *perfringens* and *B*. *fragilis*
.
(DOCX)

S6 File Other affected genes. (DOCX)
