# Supplementary material for: The Gut Bacterium Bacteroides thetaiotaomicron Influences the Virulence Potential of the Enterohemorrhagic Escherichia coli O103:H25
Source: PLoS One. 2015 Feb 26;10(2):e0118140. doi: 10.1371/journal.pone.0118140 (PMC4342160; doi:10.1371/journal.pone.0118140)
Supplement: S1 File — Summary of changes in expression of selected categories of genes in microarray analysis of EHEC NIPH-11060424 in co-culture with B. thetaiotaomicron relative to EHEC NIPH-11060424 in pure culture. (DOCX) [file pone.0118140.s001.docx]

| ***Category/***  ***Gene symbol*** | ***Gene ID*** | ***Gene description*** | ***OD=0.5***  ***log_2_ratio*** | |
| --- | --- | --- | --- | --- |
| ***LEE-genes*** |  |  | |  |
| *escS* | 8475184 | TTSS structure protein | | **3.75** |
| *escR* | 8475183 | type III secretion system protein | | **3.60** |
| *escT* | 8475185 | TTSS structure protein | | **3.51** |
| *ECO103_3635* | 8474196 | TTSS component | | **3.43** |
| *ECO103_3635* | 8475182 | TTSS component | | **3.40** |
| *ECO103_3636* | 8474197 | TTSS component | | **3.24** |
| *ECO103_3637* | 8474198 | TTSS component | | **3.19** |
| *grlA* | 8474871 | positive regulator | | **3.16** |
| *grlR* | 8475546 | negative regulator | | **3.04** |
| *escU* | 8474195 | TTSS structure protein | | **2.62** |
| *ECO103_3622* | 8475195 | putative TTSS component | | **2.37** |
| *escJ* | 8474194 | TTSS structure protein | | **2.31** |
| *espZ* | 8476047 | TTSS secreted effector | | **2.12** |
| *ECO103_2063* | 8473565 | TTSS secreted effector NleG-like protein | | 1.87 |
| *escC* | 8476769 | TTSS structure protein | | 1.77 |
| *ECO103_2223* | 8478399 | TTSS secreted effector NleF-like protein | | 1.75 |
| *ECO103_2292* | 8473703 | TTSS secreted effector EspO-homolog | | 1.74 |
| ***cit-operon*** |  |  | |  |
| *citD* | 8474900 | citrate lyase subunit gamma | | **4.5** |
| *citC* | 8474899 | citrate lyasesynthetase | | **4.18** |
| *citG* | 8474903 | triphosphoribosyl-dephospho-CoA synthase | | **3.85** |
| *citT* | 8476579 | citrate/succinate antiporterCitT | | **2.97** |
| *citE* | 8474901 | citrate lyase. citryl-ACP lyase subunit | | **2.72** |
| ***Metabolic genes*** |  |  | |  |
| *glpD* | 8475489 | glycerol-3-phosphate dehydrogenase | | **-2.95** |
| *trxC* | 8477840 | thioredoxin 2 | | **-2.80** |
| *fixC* | 8475284 | oxidoreductase | | **-2.74** |
| *fixX* | 8475285 | 4Fe-4S ferredoxin-type protein | | **-2.59** |
| *speF* | 8476360 | ornithine decarboxylase isozymeSpeF. inducible | | **-2.53** |
| *hemF* | 8474654 | coproporphyrinogen III oxidase | | **-2.34** |
| *hmp* | 8475515 | nitric oxide dioxygenase | | **2.30** |
| *hcp* | 8475583 | hydroxylamine reductase | | **2.28** |
| *srlE* | 8476837 | glucitol/sorbitol-specific enzyme IIB component | | **-2.27** |
| *srlA* | 8476018 | glucitol/sorbitol-specific enzyme IIC component of PTS | | **-2.25** |
| *fixB* | 8475283 | putative electron transfer flavoproteinFixB | | **-2.18** |
| *hyuA* | 8477904 | D-stereospecific phenylhydantoinase | | **-2.10** |
| *ECO103_5177* | 8474511 | putative serine/threonine kinase | | **2.08** |
| *bcp* | 8475444 | thioredoxin-dependent thiol peroxidase | | **2.06** |
| *aes* | 8475598 | acetyl esterase | | **-2.05** |
| *hcr* | 8476366 | HCP oxidoreductase. NADH-dependent | | **2.04** |
| *dsdA* | 8473987 | D-serine ammonia-lyase | | **-2.03** |
| *tnaA* | 8476946 | tryptophanase | | **-2.02** |
| *mdh* | 8475058 | malate dehydrogenase | | 1.97 |
| ***Stress response*** |  |  | |  |
| *yjiX* | 8478262 | hypothetical protein | | **5.00** |
| *yjiY* | 8478276 | putative inner membrane protein | | **3.74** |
| *cspA* | 8475637 | major cold shock protein | | **2.96** |
| *yjiA* | 8474488 | putative GTP-binding protein | | **2.81** |
| *cspG* | 8474963 | Cold shock protein | | 1.95 |
| *dinQ* | 8475537 | damage inducible protein | | 1.89 |
| ***Cell membrane/cell wall*** |  |  | |  |
| *ytfE* | 8474984 | regulator of cell morphogenesis and cell wall metabolism | | **2.77** |
| *ECO103_3792* | 8474267 | putative membrane-associated. metal-dependent hydrolase | | **2.29** |
| ***Transporters*** |  |  | |  |
| *potE* | 8476302 | putrescine transporter | | **-3.21** |
| *yaaU* | 8475286 | transporter | | **-2.12** |
| *mntH* | 8473988 | manganese/divalent cation transporter | | **-2.08** |
| *tnaB* | 8475956 | low affinity tryptophan transporter | | **-2.07** |
| *tonB* | 8477474 | membrane spanning protein | | **-2.00** |
| *tehA* | 8477515 | potassium-telluriteethidium and proflavin transporter | | 1.92 |

**boldface values represent significant changes (P≤0.05)*
