# Supplementary material for: The Gut Bacterium Bacteroides thetaiotaomicron Influences the Virulence Potential of the Enterohemorrhagic Escherichia coli O103:H25
Source: PLoS One. 2015 Feb 26;10(2):e0118140. doi: 10.1371/journal.pone.0118140 (PMC4342160; doi:10.1371/journal.pone.0118140)
Supplement: S2 File — Summary of changes in expression of genes of selected categories in EHEC NIPH-11060424 cultured in spent medium from B. thetaiotaomicron relative to pure culture. (DOCX) [file pone.0118140.s002.docx]

| ***Category/*** | ***Gene ID*** | ***Gene description*** | ***OD=0.5*** |
| --- | --- | --- | --- |
| ***Gene symbol*** |  |  | ***log_2_ratio*** |
| ***Motility/chemotaxis*** |  |  |  |
| *cheY* | 8474890 | chemotaxis regulatory protein | **3.32** |
| *cheZ* | 8475307 | chemotaxis regulator | **3.26** |
| *cheR* | 8474886 | chemotaxismethyltransferase | **3.14** |
| *cheB* | 8474889 | chemotaxis-specific methylesterase | **3.06** |
| *tar* | 8476885 | methyl-accepting chemotaxis protein II | **2.65** |
| *motB* | 8474885 | flagellar motor protein | **2.61** |
| *tap* | 8474887 | methyl-accepting protein IV | **2.58** |
| *cheW* | 8474834 | purine-binding chemotaxis protein. cheW | **2.58** |
| *yhjH* | 8478074 | yhjH EAL domain containing protein involved in flagellar function | **2.44** |
| *fliS* | 8475313 | flagellar protein | **2.43** |
| *fliT* | 8475328 | flagellar biosynthesis protein | **2.36** |
| *cheA* | 8474888 | chemotaxis protein | **2.31** |
| *motA* | 8476045 | flagellar motor protein | **2.15** |
| *flgK* | 8475301 | flagellar hook-associated protein | **1.96** |
| *lrhA* |  | DNA-binding transcriptional repressor LrhA of flagellar. motility and chemotaxis genes | -1.7 |
| ***cit-operon*** |  |  |  |
| *citC* | 8474899 | citrate lyasesynthetase | **4.54** |
| *citD* | 8474900 | citrate lyase subunit gamma | **4.11** |
| *citG* | 8474903 | triphosphoribosyl-dephospho-CoA synthase | **4.03** |
| *citE* | 8474901 | citrate lyase. citryl-ACP lyase subunit | **2.72** |
| *citX* | 8474902 | 2'-(5''-triphosphoribosyl)-3'-dephospho-CoA:apo- citrate lyase | **2.65** |
| *citT* | 8476579 | citrate/succinate antiporter | **2.28** |
| ***Other metabolic genes*** |  |  |  |
| *speF* | 8476360 | ornithine decarboxylase | **-4.21** |
| *lamB* | 8475913 | lamB maltose outer membrane porin | **-4.04** |
| *malM* | 8475811 | maltose regulonperiplasmic protein | **-3.49** |
| *adiA* | 8474613 | biodegradative arginine decarboxylase AdiA | **3.44** |
| *otsB* | 8476239 | trehalose-6-phosphate phosphatase | **3.42** |
| *sdaB* | 8476752 | L-serine deaminase II | **-2.91** |
| *otsA* | 8477658 | trehalose-6-phosphate synthase | **2.89** |
| *sufC* | 8476870 | cysteine desulfurase ATPase component | **2.83** |
| *cadA* | 8478227 | lysine decarboxylase 1 | **-2.79** |
| *tdcB* | 8476900 | threonine dehydratase | **-2.58** |
| *tnaA* | 8476946 | tryptophanase | **-2.52** |
| *sufA* | 8476868 | iron-sulfur cluster assembly scaffold protein | **2.51** |
| *sufD* | 8476873 | cysteine desulfurase activator complex subunit | **2.38** |
| *dsdA* | 8473987 | D-serine dehydratase | **-2.37** |
| *fixC* | 8475284 | putative oxidoreductase | **-2.22** |
| *sufS* | 8476871 | bifunctional cysteine desulfurase/selenocysteinelyase | **2.2** |
| *srlE* | 8476837 | glucitol/sorbitol-specific enzyme IIB component | **-2.16** |
| *gadA* | 8475413 | glutamate decarboxylase A. PLP-dependent | **2.15** |
| *sufB* | 8476869 | cysteine desulfurase activator complex subunit | **2.14** |
| *astB* | 8474770 | succinylargininedihydrolase | **-2.08** |
| *malQ* | 8475533 | 4-alpha-glucanotransferase | **-2.08** |
| *astD* | 8474767 | succinylglutamicsemialdehyde dehydrogenase | **-2.07** |
| *srlA* | 8476018 | glucitol/sorbitol-specific enzyme IIC component of PTS | **-2.05** |
| *talA* | 8475907 | transaldolase A | **2.05** |
| *ECO103_1575* | 8477526 | putative acetyltransferase | **2.03** |
| *ECO103_5185* | 8474519 | putative exclusion protein | **-2** |
| *poxB* | 8475889 | pyruvate dehydrogenase | **2** |
| ***Stress response*** |  |  |  |
| *yjiX* | 8478262 | hypothetical protein | **2.63** |
| *yjiY* | 8478276 | putative inner membrane protein | 1.8 |
| ***Cell membrane/cell wall*** |  |  |  |
| ompF | 8474760 | outer membrane protein F | -2 |
|  |  |  |  |
| ***Transporters*** |  |  |  |
| potE | 8476302 | putrescine transporter | -4.50 |
| proV | 8476180 | glycine betaine transporter ATP-binding subunit | 4.32 |
| proW | 8476407 | glycine betaine transporter membrane protein | 4.18 |
| proX | 8476408 | glycine betaine transporter periplasmic subunit | 4.11 |
| malK | 8475909 | maltose/maltodextrin transporter ATP-binding protein | -3.6 |
| cadB | 8474836 | lysine/cadaverine antiporter | -3.23 |
| malE | 8475910 | maltose ABC transporter periplasmic protein | -2.92 |
| sdaC | 8477886 | putative serine transporter | -2.48 |
| xylF | 8477131 | D-xylose transporter subunit | -2.4 |
| tnaB | 8475956 | low affinitytryptophan transporter | -2.39 |
| tdcC | 8476901 | threonine/serine transporter | -2.2 |
| ydhC | 8477564 | inner membrane transport protein YdhC | 2.12 |
| yqcE | 8477883 | putative transporter | -2.1 |
| yhdW | 8478015 | putative periplasmic binding transport protein | -1.98 |
| yhdX | 8478016 | putative transport system permease protein | -1.98 |
| glcA | 8474210 | glycolate transporter | -1.96 |
| garP | 8475428 | putative (D)-galactarate transporter | -1.95 |

**boldface values represent significant changes (P≤0.05)*
