# Supplementary material for: The Gut Bacterium Bacteroides thetaiotaomicron Influences the Virulence Potential of the Enterohemorrhagic Escherichia coli O103:H25
Source: PLoS One. 2015 Feb 26;10(2):e0118140. doi: 10.1371/journal.pone.0118140 (PMC4342160; doi:10.1371/journal.pone.0118140)
Supplement: S3 File — Summary of changes in expression genes in microarray analysis of EHEC NIPH-11060424 in co-culture with B. thetaiotaomicron and spent medium relative to when EHEC NIPH-11060424 is cultured alone (3 hours after induction with MMC). (DOCX) [file pone.0118140.s003.docx]

| **Category/** | **Gene ID** | **Gene description** | **3 hrs after ind./** |
| --- | --- | --- | --- |
| **Gene symbol** |  |  | **log_2_ratio** |
| **in co-culture** |  |  |  |
| *ECO103_2069* | 8473571 | tail assembly protein | -4.23 |
| *stx2a* | 8473941 | Shiga toxin 2 subunit A | -4.04 |
| *ECO103_5159* | 8478281 | integrase | -3.81 |
| *ECO103_5203* | 8474537 | endolysin | -3.75 |
| *ECO103_2839* | 8473935 | antirepressor | -3.73 |
| ECO103_0518 | 8473152 | phage regulatory protein CII | -3.5 |
| *ECO103_1197* | 8473307 | endolysin | -3.3 |
| stx2b | 8473940 | Shiga toxin 2 subunit B | -3.23 |
| *ECO103_0533* | 8473167 | lipoprotein Bor | -3.11 |
| *ECO103_2326* | 8473737 | endolysin | -3.01 |
| *ECO103_0794* | 8473244 | minor tail protein | -2.96 |
| *ECO103_5181* | 8474515 | antirepressor protein Cro | -2.94 |
| *ECO103_5212* | 8474546 | head protein/prohead protease | -2.94 |
| *ECO103_2846* | 8475737 | late gene regulator Q | -2.83 |
| *ECO103_2325* | 8473736 | antirepressor | -2.71 |
| *ECO103_2850* | 8473946 | helicase | -2.29 |
| *recX* | 8474632 | regulatory protein RecX for RecA | -2.09 |
| *ECO103_2864* | 8473960 | exonuclease | -2.04 |
| **in spent medium** |  |  |  |
| *stx2a* | 8473941 | Shiga toxin 2 subunit A | -4.16 |
| *stx2b* | 8473940 | Shiga toxin 2 subunit B | -3.61 |
| *ECO103_2878* | 8473974 | antirepressor | -3.42 |
| *ECO103_0533* | 8473167 | lipoprotein Bor | -3.33 |
| *ECO103_2069* | 8473571 | tail assembly protein | -3.04 |
| *ECO103_1197* | 8473307 | endolysin | -2.79 |
| *ECO103_2326* | 8473737 | endolysin | -2.7 |
| *ECO103_2325* | 8473736 | antirepressor | -2.67 |
| *ECO103_0794* | 8473244 | minor tail protein | -2.64 |
| *ECO103_2864* | 8473960 | exonuclease | -2.53 |
| *ECO103_2846* | 8475737 | late gene regulator Q | -2.49 |
| *ECO103_2850* | 8473946 | helicase | -2.33 |
| *ECO103_5212* | 8474546 | head protein/prohead protease | -2.3 |
