# Supplementary material for: The Gut Bacterium Bacteroides thetaiotaomicron Influences the Virulence Potential of the Enterohemorrhagic Escherichia coli O103:H25
Source: PLoS One. 2015 Feb 26;10(2):e0118140. doi: 10.1371/journal.pone.0118140 (PMC4342160; doi:10.1371/journal.pone.0118140)
Supplement: S4 File — (DOCX) [file pone.0118140.s004.docx]

|  | PRIMER SEQUENCES |  |  |  |
| --- | --- | --- | --- | --- |
| Gene | Forward (5' to 3') | Reverse (5' to 3') | Slope^a^ | % Eff^b^ |
| *gapA* | AGGTCTGATGACCACCGTTC | AACGGTCAGGTCAACTACGG | -3.3 | 99.7 |
| *grlA* | GCCGAAGCATTCGGTATAAA | TTTTCTTTTTGGTCCGGTTG | -3.3 | 99.5 |
| *escU* | CCGTCGCCTTCATTCTGAGA | GATCACGGTTGATTTTTTGATGTTA | -3.2 | 92.1 |
| *Ler* | ATGTGCCTGATGATGGACTC | AGGTCTGCCCTTCTTCATTG | -3.1 | 88 |
| *escJ* | TAGCACCATCGGTCATTCAG | ACATATTACCCGTCCTGTCCTG | -3.2 | 84 |
| *escV* | CGCACTAATGCTCGTAATGG | TTCCAAGCTATCCCCAACAG | -3.2 | 88 |
| *escF* | AACGCTGAGTGATTCTGTGC | AATATTGCTGAACCGCGAAC | -3.4 | 97.8 |
| *espA* | CGCTTGAGCTGAAATAGCTG | AACGCTGAGTGATTCTGTGC | -3.4 | 95 |
| *eae* | ACATTATGGAACGGCAGAGG | AAGCGGGAGTCAATGTAACG | -3.1 | 88 |
| *tir* | ATCAACAGCTTCCAGCGTTC | TTGTAGGATCATCCGGTTCC | -3.2 | 96 |
| *cheY* | CAGGCGGTTATGGATTTGTT | AATGGCTTCACCACATAGCC | -3.3 | 99.6 |
| *cheZ* | AAATCGTCCACCTGATCCTG | AGTTGCTGATGGTGCTGTTG | -3.7 | 86.7 |
| *cheB* | TGGTAATTTGCGCCACTACG | GGTTAAAGAAGCCGAAGACG | -3.3 | 99.3 |
| *motA* | GCTGGAATAGAGCGTTTTGC | ATGCAGTGCGTCAAAGTCAC | -3.3 | 97.4 |
| *motB* | CAGGGGGAAGTGAATAAGCA | TTCTAAACATCGGGCGATTC | -3.1 | 91 |
| *citD* | TGAGTCTGGGGATGTGATGA | CTTCCAGTCGTGCACGTAAA | -3.6 | 90 |
| *glpD* | CATCAATGGTGCTGGTATCG | GTTTTGAACTGGCGGAAGAG | -3.1 | 90.4 |
| *trxC* | CGTTTGTACCCATTGTCAGG | TCTCCGTCAAACAAGTCGTG | -3.3 | 83 |
| *potE* | AACCAAGGAAGGTCACGATG | TCAGAAGGTCGCTAATGTGC | -3.6 | 91 |

^a^Slope was calculated from the regression line in the standard curve

^b^Efficiency was calculated using the slope of the regression line in the standard curve
