# Supplementary material for: The Gut Bacterium Bacteroides thetaiotaomicron Influences the Virulence Potential of the Enterohemorrhagic Escherichia coli O103:H25
Source: PLoS One. 2015 Feb 26;10(2):e0118140. doi: 10.1371/journal.pone.0118140 (PMC4342160; doi:10.1371/journal.pone.0118140)
Supplement: S6 File — (DOCX) [file pone.0118140.s006.docx]

**Other affected genes**

The presence of *B. thetaiotaomicron* did not result in a major stress response for the EHEC strain. However, some genes involved in stress responses including cold shock genes, as well as genes involved in cell wall- and cell membrane synthesis were up-regulated in EHEC NIPH-11060424 grown in co-culture or in the presence of spent medium from *B. thetaiotaomicron* relative to pure culture.
